# Bibliographical

**Published:** 1872-10

**Authors:** 


### EDITORIAL AND MISCELLANEOUS.

### BIBLIOGRAPHICAL.

*A System of Surgery—Pathological, Diagnostic, Therapeutic and Operative. By* SAMUEL D. GROSS, M.D., LL.D., D.C.L., Oxon, Professor of Surgery in the Jefferson Medical College, of Philadelphia, etc. Illustrated by upwards of fourteen hundred engravings. Fifth Edition, greatly enlarged and thoroughly revised. In two volumes. Philadelphia: Henry C. Lea. 1872.

Through the kindness of the publishers these two magnificent volumes have been placed upon our table. This great work of Professor Gross is so well known to the profession, both in Europe and America, that anything like an extended review upon our part would be a work of supererogation. We desire, however, to say a few words in regard to this, the fifth, and as we learned from the distinguished author himself, the last revised edition, as his active literary labors in the way of book-making terminated with the completion of this edition.

In many particulars this edition, as compared with the first, is a new book, as many of the chapters have been entirely rewritten and all carefully revised. From a hasty review of the work, it is made evident that the great object of the learned author in this edition was to place upon record the exact status of the subjects treated at the date of the edition. That he has had the the manliness to set aside long entertained and favorite theories for those, the result of more recent research, is evinced by the many changes in the text of the work. No one, perhaps, has succeeded so well as Dr. Gross, in writing at once a college text-book and a book of reference for the established practitioner. By common consent, it is admitted to be one of the most thorough practical treatises in the English language on surgery in general, and certainly the best book of reference for the general practitioner. No physician's library will be complete without "Gross' Surgery."

*Ovarian Tumors: Their Pathology, Diagnosis, and Treatment Especially by Ovariotomy.* By RANDOLPH PEASLEE, M.D., LL.D., Professor of Gynæcology in the Medical Department of Dartmouth College; Attending Surgeon of the New-York Woman's Hospital; President of the New-York Academy of Medicine, etc. With Fifty-six Illustrations on Wood. D. Appleton & Co., 549 Broadway, New-York.

This work was undertaken from a conviction, as stated by the author, that a practical treatise in the English language, upon the subject of which it treats, is greatly needed. While a number of writers have given their individual experience as ovariotomists, no work has appeared of broader scope, which proposes to cover the whole ground, so far as practicable within the limits of a single volume. The author's experience for twenty-five years past is supposed to qualify him to impart some information of value.

The first part of the work includes the normal anatomy of the ovary, and the pathological anatomy; the pathology, diagnosis and treatment of ovarian tumors, excepting by ovariotomy. The second part is devoted to ovariotomy alone—including its history, statistics, practical details, and after-treatment. It aims to decide all practical questions by the aggregate experience of all ovariotomists up to the present time.

Such a work, from so distinguished an author and so skillful a surgeon and gynæcologist, ought to command the attention of all who feel any interest in the subjects treated.

*Hysterology. A Treatise, Descriptive and Clinical, on the Diseases and Displacements of the Uterus* By EDWIN NESBIT CHAPMAN, M.A., M.D., Late Professor of Obstetrics, Diseases of Women and Children, and Clinical Midwifery, in the Long-Island College Hospital. William Wood & Co., Publishers, New-York.

We have been interested in the examination of this work beyond what is usual with us, especially on account of the novelty of the views presented.

The body of the work is the reproduction of the author's lectures on the diseases and displacements of the uterus, in the Long-Island College Hospital. The *cliniques* that present the data from which the doctrines inculcated have been deduced, are represented by cases and commentaries, thus presenting both a somewhat systematic as well as clinical treatise on what the author calls "Hysterology," as a more distinctive title of a monograph on uterine disease than "Gynæcology"—the latter embracing all the diseases peculiar to females.

*A Year-Book of Therapeutics, Pharmacy, and Allied Sciences.* Edited by HORATIO C. WOOD, JR., M.D. William Wood & Co., New-York.

This is a bound volume of that useful little periodical which is published by the above house under the name of "New Remedies." It should be read by all who would keep abreast with the progress of the most progressive of all the sciences.

*The Science and Practice of Medicine.* By WILLIAM AITKEN, M.D., Edinburgh, Professor of Pathology in the Army Medical School. Third American from sixth London Edition, greatly enlarged, remodeled, carefully revised, and many portions rewritten, adopting the new Nomenclature, and following the order of Classlification of Diseases, published by the Royal College of Physicians, of London, with additions by MEREDITH CLYMER, M.D., Ex-Professor of the Institutes and Practice of Medicine in the University of of New York, formerly Physician to the Philadelphia Hospital, etc. In two volumes, with steel plate map and one hundred and eighty wood-cuts. Lindsay & Blakiston, Publishers, Philadelphia.

This is the sixth edition of Aitken, and in the period of fourteen years since the first edition was issued, it has maintained a high position as a representative book of medical science and practice, representing as it does, not only the views of its distinguished author, but those of the most prominent and able men in the profession.

The author informs us that during the eighteen months preceding its issue, he was engaged in a careful revision of the whole work, which had been out of print nearly twelve months. The plan of the work has been again remodeled so as to embrace a consideration of the topics in the order of the classification of the College of Physicians, of London, whose nomenclature was adopted in the last edition, thereby tending to remove the difficulties which arise from the complexity and indefinitness of medical terminology. Compared with the previous edition, the new material added in the present, by the author, is equivalent in bulk to a third volume added to the last edition. The work has indeed been, to a great extent, re-written, and descriptions of many diseases altogether omitted in former editions, are now introduced. The diagrams illustrative of the typical ranges of body temperature in febrile diseases (which were given for the first time in a text book in the third edition of this work, in 1863), have been carefully reconsidered, together with the whole of this important and practically useful subject now so generally adopted. The result has been, that the diagrams have been nearly all redrawn, and cut upon an improved model. Additional wood-cuts have been also introduced, rendering the descriptions in the text more intelligible.

The American edition is ably edited by Dr. Clymer, who has articles (besides having a large portion of his editorial matter in the last American edition, incorporated in the body of the work) on thirteen distinct diseases, viz: camp measles, spinal symptoms in typhoid fever, typho-malarial toxemia, etc.

*The Treatment of Syphilis with Subcutaneous Sublimate Injections.* By Dr. Geo. Levin, Professor at the T. R. Wilk University and Surgeon in Chief of the Syphilitic Ward and Skin Diseases of the Charité Hospital, Berlin. By Carl Proegler, M.D., late Surgeon in the Prussian service and in the United States Army, and E. H. Gale, M.D., late Surgeon in the United States Army. Lindsay & Blakiston, Publishers.

As an indication of the character of the work, we present the views contained in the translators' and author's preface. The translators say, that one having witnessed the successful workings of the subcutaneous use of sublimate in the cure of syphilis at European hospitals, and both having had pleasing results in private practice, together with the well-known standing of Professor Levin in all matters pertaining to syphilitic diseases, they have been induced to translate the result of his experiments in the venereal wards of the Royal Charité Hospital, Berlin, and lay them before their American readers. While they do not think the hypodermic method is the *only* way to eradicate syphilis, still, in many cases, they think it best, inasmuch as it is speedy and sure. Besides, they suggest that all who are opposed to introducing large quantities of mercury into the system, can not fail to endorse this plan, if the drug is to be used at all, as from one and a half to two and a half grains of sublimate, on an average, are sufficient for a cure. The author informs us that nearly seven years have elaspsed since he introduced the subcutaneous sublimate injection into the syphilitic wards under his charge at the Royal Charité Hospital, Berlin, and during this time he has treated, by this method, over two thousand patients, including those belonging to his private practice. He gives the following statistical data which speak for themselves. In Berlin the law exists that every woman given to prostitution may be under *police surveillance*, and those who lead a suspicious life, have to be examined weekly, and any exploration giving a warrantable proof of syphilis, gives the surgeon the right to send them to his syphilitic ward, all of which women have been treated by himself exclusively since March, 1865, by this new method, numbering about fourteen hundred. At the end of July, 1869, there had not been more than twenty female patients who had returned to his ward on account of syphilitic relapses, all of which were of a very slight character. Notwithstanding, however, such remarkable results he states his unwillingness to be regarded as adopting the view that the injection method is the only cure to be adopted. It is an interesting field of inquiry and well worthy the attention of the profession.

***Functional Diseases of the Renal, Urinary and Reproductive Organs, with a General Review of their Urinary Pathology.*** **By D. Campbell Black, M.D., Edinburgh, Author of "Observations on Therapeutics and Disease," "On Certain Points in the Pathology and Treatment of Gonorrhœa," and "Syphilitic and Phagedenic Ulceration." Lindsay & Blakiston, Publishers, Philadelphia.**

The author discusses, in seven chapters and two hundred and eighty-six pages, "The Conditions that Affect the Secretion of Urine, with Special Reference to Suppression;" "Retention of Urine—its Varieties, Causes and Treatment;" "Irritable Bladder and Strangury;" "The Pathology and Treatment of Nocturnal Enuresis and Spermatic Incontinence;" "Sterility in the Male;" "Male Impotency," and "Anomalous Urethral Discharges;" with an appendix on quack advertisements in regard to spermatorrhea. This is an interesting and valuable discussion of a number of subjects upon which the profession greatly needs enlightenment.

Finally, we have a little book of fifty-nine pages, from the same publishers, by Meredith Clymer, M.D., on "Cerebro-Spinal Meningitis," with an appendix on some points as regards the causes of the disease as shown by the history of the present epidemic in the city of New York. We welcome anything coming from so intelligent a source upon so obscure a subject as the history, geographical and clinical pathology and treatment of epidemic cerebro-spinal meningitis. The sketch was originally written in 1866, revised in 1868, and corrected in 1872 in connection with the prevalence of the disease in the city of New York. While it does not claim to be a complete monograph upon the subject, the author believes that it will throw some light upon the vexed question of the cause of the disorder.

The various medical works noticed in this JOURNAL, or any other medical book, can be furnished by Messrs. Richards & Co., and Messrs. Lynch & Co., of this city.

